# Rumen Microbial Composition and Fermentation Variables Associated with Methane Production in Italian Simmental Dairy Cows

**DOI:** 10.3390/ani16030510

**Published:** 2026-02-05

**Authors:** Cristina Pavanello, Marcello Franchini, Alberto Romanzin, Lara Tat, Stefano Bovolenta, Mirco Corazzin

**Affiliations:** Department of Agricultural, Food, Environmental and Animal Sciences, University of Udine, Via delle Scienze 206, 33100 Udine, Italy; pavanello.cristina@spes.uniud.it (C.P.); marcello.franchini@uniud.it (M.F.); lara.tat@uniud.it (L.T.); stefano.bovolenta@uniud.it (S.B.); mirco.corazzin@uniud.it (M.C.)

**Keywords:** methane emissions, rumen microbiome, methanogen diversity, dairy cows, microbial efficiency

## Abstract

The study investigated differences in ruminal and fecal microbiota composition, fermentation traits, and volatile organic compounds (VOC) in Simmental dairy cows classified as high (HME) or low (LME) methane emitters. Methane emissions from 48 cows were quantified using the Laser Methane Smart portable gas detector. The 12 animals with the highest and lowest emissions were selected and assigned to the HME and LME groups, respectively, balanced for body weight, days in milk, and body condition score. Rumen fluid and fecal samples were analyzed for pH, ammonia, volatile fatty acids (VFA), VOC, and microbiota composition. As expected, CH_4_ emissions were significantly higher in HME than in LME cows (22.5 vs. 13.2 g/kg DMI; 16.9 vs. 8.4 g/kg FCM). The neutral detergent fiber digestibility was higher in HME cows (51.4% vs. 47.9%). The valeric acid concentration and the acetate-to-propionate ratio were significantly higher in HME cows (3.53 vs. 3.31). The VOC profiles significantly differed between groups in both feces and rumen fluid. The microbiota analysis revealed a significant difference between groups at the order and genus levels (Bray–Curtis dissimilarity). The Shannon index was higher in LME cows (2.08 vs. 1.95). HME cows exhibited a higher abundance of *Methanosphaera* and Methanobacteriales. Overall, the results indicate that re-shaping the rumen microbial community can play a key role in reducing methane emissions, strengthening the case for microbiome-driven approaches and offering insights that can support mitigation strategies across dairy production systems.

## Simple Summary

This study aims to determine the differences in ruminal and fecal microbiota between lactating dairy cows that are considered high and low methane emitters. The emissions of 48 dairy cows were measured using a hand-held methane sensor during feeding. To assess the differences in methane emissions, the 12 highest and 12 lowest emitters were selected, and rumen fluid and fecal samples were taken in order to analyze the volatile fatty acids and the volatile organic compounds. As expected, the grams of methane emitted per kg of dry matter intake were higher in the high emitters than in the low emitters. The high emitters showed a less diverse microbiome dominated by methanogens, whereas low emitters had a more diverse community dominated by hydrogen-utilizing bacteria that reduce hydrogen availability for methanogenesis.

## 1. Introduction

In livestock agri-food systems, methane (CH_4_) emissions are strongly influenced by agronomic practices, such as manure management, feed production, and animal husbandry. The most significant source of CH_4_ is enteric fermentation, which accounts for 46.8% of total greenhouse gas (GHG) emissions from livestock agri-food systems and 5.6% of global GHG, respectively [1]. Thus, dairy cattle farming is particularly relevant, because roughly 95% of enteric CH_4_ emissions are produced in the rumen [2].

In recent years, several strategies have been developed to mitigate enteric CH_4_ emissions from dairy cows, mainly targeting dietary modification, such as adjusting the forage-to-concentrate ratio or supplementing with algae, oils, tannins, or commercial additives. Genetic selection has also been attempted as a mitigation strategy [2]. In this context, several studies have been undertaken; however, the results obtained do not provide a clear picture. Kjeldsen et al. [3] classified dairy cows as high or low CH_4_ emitters and observed that low emitters had lower organic matter (OM) digestibility, despite emissions not being linked to rumen size. Stepanchenko et al. [4] similarly reported reduced OM and fiber digestibility in low emitters, who also tended to be smaller than high emitters. However, Terranova et al. [5] questioned whether CH_4_ output truly reflects the amount of digestible fiber.

Diet-driven shifts in rumen microbiota also contribute to CH_4_ variation. Low-emitting cows often show higher abundances of *Succinivibrio*, organisms that compete for hydrogen (H_2_) and favor propionate formation [6], along with lower levels of *Methanobrevibacter* [7]. Because Succinivibrionaceae thrive in starch-rich diets [4], microbial responses to feed composition can substantially influence methanogenesis. Still, research connecting rumen fermentation to CH_4_ production remains inconsistent [8,9]. The microbial network that governs H_2_ use and regulates CH_4_ formation is complex [10]. More clarity is needed on how fermentation patterns, microbial composition, and animal performance interact [4].

Another key determinant of CH_4_ production is the animal’s genetic background. Specific heritable microbiome traits are related to the cow’s feed efficiency, which in turn affects the fermentation efficiency and CH_4_ production [11]. Studies on breeds such as Jersey cattle show that distinct microbial profiles are associated with lower CH_4_ emissions under controlled dietary conditions [7]. Similarly, genetic crosses, such as those between Holstein–Friesian and Jersey, have further demonstrated that the genetic background can influence the rumen microbiota and CH_4_ production [12]. To the knowledge of the authors, studies on the rumen microbiome of Italian Simmental dairy cows are extremely limited, with only one previous investigation comparing Italian Simmental and Holstein cows using whole-genome shotgun sequencing [13].

Improving our understanding of how microbiome composition and CH_4_ production are interconnected opens the door for targeted interventions, such as the possibility of favoring low CH_4_-emitting microbial profiles. The aim of this study was to investigate the differences between high methane-emitting (HME) and low methane-emitting (LME) dairy cows of the Italian Simmental breed, focusing on the relationship between the microbial diversity and composition in rumen and feces, volatile organic compounds (VOC), and rumen fermentation variables. By analyzing the microbiota composition and its link to CH_4_ emissions, this study seeks to inform future strategies to enhance the energy efficiency of rumen fermentation in dairy cows through a reduction in CH_4_ emissions.

## 2. Materials and Methods

### 2.1. Animals and Sampling

Forty-eight Italian Simmental dairy cows (*n* = 24 primiparous and *n* = 24 multiparous) were selected from a herd of 420 cows, and their CH_4_ emissions were measured as described below. Two groups were subsequently created based on CH_4_ emissions: the HME group (n = 12; 489.2 (254.0) g/day; median (interquartile range)) and the LME group (n = 12; 309.1 (59.2) g/day). The two groups were balanced and similar for body condition score (BCS, 3.63 (0.75) points; *p* > 0.05; Ref. [14]), days in milk (DIM, 171.5 (47.8) days; *p* > 0.05), and body weight (BW; 699.0 (90.8) kg; *p* > 0.05). Each group consisted of 6 primiparous and 6 multiparous dairy cows.

Animals were herd reared and fed a total mixed ratio (TMR), as reported in Table 1, and the same diet was given to the animals for over a month before the CH_4_ measurement. The DMI was estimated following de Souza et al. [15].

On the same day, 500 mL of rumen fluid was collected from each of the 24 cows via trans-esophageal probe (Bovivet stomach probang system mark II, Kruuse, Langeskov, Denmark), discharging the first 150 mL to avoid saliva contamination. Approximately 1 kg of feces was collected through grab sampling. The samples were taken while the animals were re-strained using a head gate. Samples were collected once per cow at the end of the methane measurements phase, after milking, at a time when the animals were not eating. The goal was to determine the pH, ammonia (NH3) concentration, volatile fatty acids (VFA), and VOC. Rumen fluid samples were also used for microbial DNA extraction to perform metagenomics analysis, while feces samples were also used to determine the diet digestibility. Furthermore, the TMR was sampled immediately after the feed distribution following the technique found in Robinson et al. [17]. Furthermore, the feeding behavior and milk yield were recorded daily, and a 250 mL milk sample was collected from each animal at the morning milking. After collection, all samples were refrigerated, transported to the laboratory in less than an hour, and prepared for the analyses.

### 2.2. Methane and Feeding Behavior Measurements

To measure the CH_4_ concentration in the breath of each animal, the Laser Methane Smart (LMS; Tokyo Gas Engineering Solutions Ltd., Tokyo, Japan) portable gas detector was used, based on the measurement principles described by Chagunda et al. [18]. The measurement duration was set to 5 min per animal, 1 min longer than recommended by Boré et al. [19], at a distance of 1 m from the animal’s nostrils. Following the protocol outlined by Sorg et al. [20], two daily CH_4_ profiles were recorded for each animal on three different and consecutive days: one in the morning, immediately after the first milking, and one in the afternoon, after the second milking, both while the animals were effectively eating. CH_4_ concentrations were calculated using the arithmetic mean of all peaks recorded during the 5-min period, and daily CH_4_ emission estimates (g/day) were obtained following the method reported in Sorg et al. [20].

The feeding behavior—i.e., the eating time (ET) and rumination time (RT)—of each animal was continuously monitored using collars equipped with sensors (SenseHub Dairy, Allflex Livestock Intelligence, SCR Engineers Ltd., Netanya, Israel).

### 2.3. Laboratory Analysis

Milk composition was determined following the AOAC protocol [21]; methods 989.05, 991.20, and 896.01, and the fat corrected milk (FCM) was calculated [22].

The DM of feed samples was determined at 105 °C for 3 h and ash at 550 °C for 2 h, and the CP was calculated as N × 6.25, using the AOAC method 984.13 [21]. The NDF and ADF were analyzed without ash correction, with α-amylase pre-treatment [23]. The EE was determined using the AOAC method 920.29 [21].

The diet digestibility was determined with acid-insoluble ash (AIA) as a marker, measured in the fecal and feed samples according to Van Keulen and Young [24]. Specifically, the DM digestibility (DMd), CP digestibility (CPd), NDF digestibility (NDFd), and ADF digestibility (ADFd) were calculated as follows

Digestibility=100−(100×(% AIA in feed/% AIA in faeces)×(Faecal nutrient/% Feed nutrient))

The pH, NH3 concentration, VFA, and VOC were measured both in the rumen fluid and in the fecal samples. The pH was assessed using a pH meter (GLP 22, Crison Instru-ments, Barcelona, Spain). The concentration of NH_3_ was determined using an NH_3_ elec-trode (Ammonia Gas Sensing Combination Electrode, Hach Company, Loveland, CO, USA).

The VFA concentration was assessed, as described by Braidot et al. [25]. First, 5 mL of sample was acidified by adding 5 mL of H_2_SO_4_ 0.1 N (ratio 1:1 *v*/*v*); then, it was centrifuged at 20,000 *g* for 20 min at 4 °C. Subsequently, the supernatant was filtered using polypropylene filters (pore diameter 0.45 µm; DTO Servizi, Venice, Italy). The filtrate was injected into a high-performance liquid chromatographer with its analysis wavelength set to 220 nm. Finally, the peaks of analytes were cross-checked with the outcomes of a standard mixture.

To analyze the VOC profile, 250 μL of phosphoric acid (85%) and 100 μL of solution 3-pentanol (0.2392 g/L in water as internal standard) were added to 5 mL of rumen fluid. Next, 1.5 g of feces samples were dissolved in 5 mL of saline water (30%) and spiked with 250 μL of phosphoric acid (85%) and 100 μL of solution 3-pentanol (0.0478 g/L in water as internal standard). Glass vials (20 mL) were sealed with PTFE/silicone septa. Subsequently, the VOC profile was analyzed via SPME-GCMS composed of a GC-2030 Nexis gas chromatograph (Shimadzu, Kyoto, Japan) and a QP2020NX mass spectrometer (Shimadzu, Kyoto, Japan) and equipped with a GC auto-sampler (HTA, Brescia, Italy). The samples were preconditioned at 40 °C for 15 min, after which a 2 cm 50/30 μm divinylbenzene/carboxen/polydimethylsiloxane fiber (Supelco, Bellefonte, PA, USA) was exposed in the headspace for 15 min at the same temperature. For GC separation, a capillary column DB-Wax (30 m × 0.25 mm × 0.25 μm film thickness; Agilent Technologies, Santa Clara, CA, USA) was used with the following operating conditions: 35 °C for 5 min; then 2 °C/min to 60 °C; then 3 °C/min to 200 °C; held for 2 min; then 20 °C/min to 240 °C; with a final hold of 10 min. Injections were performed in splitless mode for 108 s, with the injection port at 250 °C. Helium was used as the carrier gas at a constant linear velocity of 37 cm/s. The mass spectrometer operated in SCAN mode (scan range *m*/*z* 25–350) with the electron impact at 70 eV, while the transfer line and ion source were kept at 240 °C at 200 °C, respectively. Volatile compounds were tentatively identified by comparing their mass spectra with those reported in the NIST spectrum library [26]. Linear retention indices were obtained from the retention times of n-alkanes and compared with those reported in the literature [26]. Relative quantities, expressed as internal standard equivalents, were calculated by integrating the areas of the chromatographic peaks in the total current.

Bacterial DNA extraction with metagenomics analysis was also performed on rumen fluid samples. Two mL of rumen fluid was collected and stored at −20 °C. The DNA extraction was carried out using the DNeasy PowerSoil Pro Kit (Qiagen, Hilden, Germany), following the manufacturer’s protocol. Bacterial community identification was conducted as described by Spanghero et al. [27]. The quality of DNA extracted was previously tested by amplifying the V1–V3 region of the 16s gene using Pro341F and Pro805R primers designed by Takahashi et al. [28]. Thereafter, the libraries were purified, amplified, normalized, mixed, loaded on an Illumina MiSeq sequencing platform, and sequenced using a paired-end 2 × 300 bp approach. Reads were filtered using a minimum read length of 250 bp, and a total of 5269 sequences were identified with an average length of 416 bp. To assign the corresponding taxonomy, the obtained ASVs were associated with the Greengenes (v. 13.8) and SILVA (v. 138) databases.

### 2.4. Statistical Analysis

Data were analyzed using the R software version 4.1.2 [29]. The effect of the CH_4_ emission group (HME vs. LME) on the animal characteristics, performance, behavior, methane emissions, nutrient digestibility, feces and rumen fermentation variables, and rumen microbial diversity and microbial composition at the order and genus taxonomic levels was assessed using a general univariate linear model, with CH_4_ emission as the fixed factor and parity (primiparous vs. multiparous) as the fixed block factor, to account for possible parity-related variability. The experimental unit was the animal.

y_ij_ =μ + CH_4_ emission group_i_ + Parity_j_ + ε_ij_

The normality of the data distribution and the homoscedasticity were evaluated using Shapiro–Wilk and Breush–Pagan tests, respectively. When the model assumptions were not met, data were log-transformed. If the assumptions were still not met after log-transformation, the same model was considered but using a robust approach. This was the case for the following variables: BCS, rumen fermentation variables: lactic acid and isobutyric acid, Shannon indices, and *Succinivibrionaceae UCG-001*. For this purpose, the robust package, version 0.7.5 was used [30], which manages heteroscedasticity and skewed distributions by computing the robust estimate with a high breakdown point and high efficiency [30]. Partial Least Squares Discriminant Analysis (PLS-DA; Ref. [31]) was used to determine the efficacy of rumen or feces VOC (X matrix) to discriminate between the HME and LME groups (Y matrix). After PLS-DA, the association networks between the rumen and feces VOC and emissions group (HME and LME) were created using the “network” function of the mix-Omics package, version 6.32.0 [32], and these are presented for descriptive purposes. The global difference between the rumen microbial communities of HME and LME was assessed using beta diversity, Bray–Curtis dissimilarity, and considering the entire microbial composition. For this purpose, permutational multivariate analysis of variance was performed considering distance matrices at both the order and genus levels, using the same model as before, with a permutation test with 100,000 permutations. The similarity between the different samples was assessed via Principal Coordinates Analysis (PCoA) (vegan package, version 2.7.1 [33]). The study addressed multiple biologically distinct hypotheses related to methane emission groups. Therefore, no global family-wise adjustment for multiple testing across all variables was applied to avoid Type II error inflation. Statistical significance was assessed at *p* < 0.05; however, given the multi-endpoint nature of the study, *p*-values should be interpreted in conjunction with biological relevance and consistency across related traits. In this text, the data are reported as the median and interquartile range.

## 3. Results

### 3.1. Milk Yield, Behavior, Diet Digestibility and CH_4_ Production

Despite the division of animals into two groups, HME and LME, no statistically significant differences were observed in the milk yield, DMI, milk composition, or feeding behavior, including the ET and RT (*p* > 0.05; Table 2). As reported in Table 2, the digestibility of the dietary components showed no statistically significant differences (*p* > 0.05), except for NDF, which was significantly higher in the HME group compared to the LME group (*p* < 0.05). The HME group showed a wider interquartile range of CH_4_ emissions (254 g/day) compared to the LME group (59.2 g/day), indicating higher variability within the high-emitting animals. As expected, the HME animals produced significantly higher CH_4_ emissions than the LME animals, both per unit of DMI (*p* < 0.01) and per unit of milk produced (*p* < 0.01; Table 2).

### 3.2. Feces and Rumen Characteristics

The characteristics of the feces and rumen fluid (i.e., pH, NH_3_, and VFA) are summarized in Table 3. The VFA in feces were similar between the HME and LME groups (*p* > 0.05), as were the NH_3_ concentrations and pH (*p* > 0.05). In the rumen fluid, the valeric acid concentration was significantly higher in HME animals than in LME animals (*p* < 0.05). Although the acetic and propionic acid concentrations did not differ between groups (*p* > 0.05), the acetic/propionic acid ratio was significantly higher in the HME group (*p* < 0.05).

**Figure 1:**
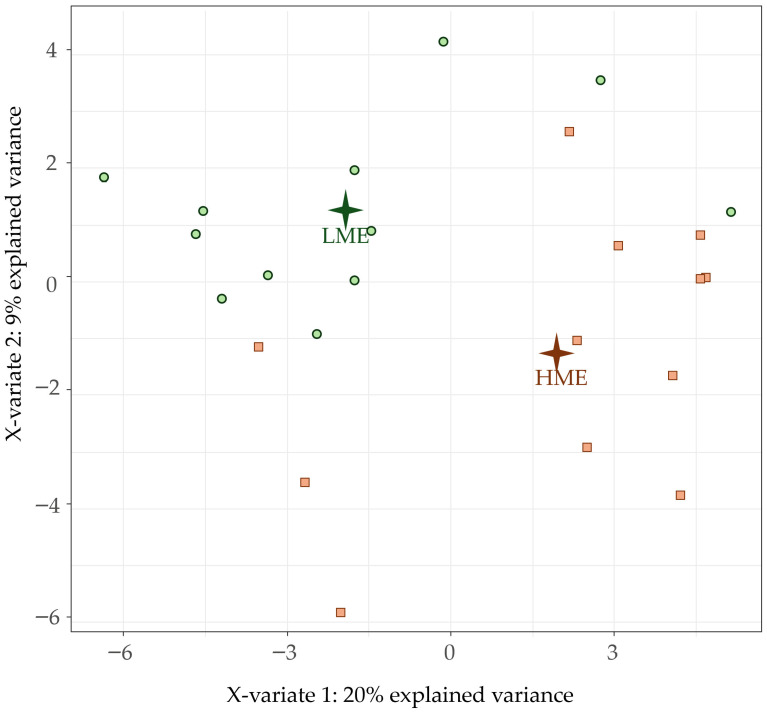
Partial Least Squares Discriminant Analysis plot of the fecal volatile organic compounds of the individual dairy cows, with separation of the two groups: high methane-emitting (HME, brown square) and low methane-emitting (LME, green circle) dairy cows.

The PLS-DA based on all detected fecal VOC is presented in Figure 1. The two principal components represented 29% of the original variance. Although the Y-matrix values showed a clear separation between the LME and HME for both components, individual animals were not completely distinguishable between the two experimental groups. The first component was positively associated with pentanal (loading: 0.18) and negatively associated with hexanoic acid (loading: −0.24) and hydrocinnamic acid (loading: −0.22). On the same component, propionic acid showed a negative association (−0.15), while acetic acid was weakly associated (−0.05).

**Figure 2:**
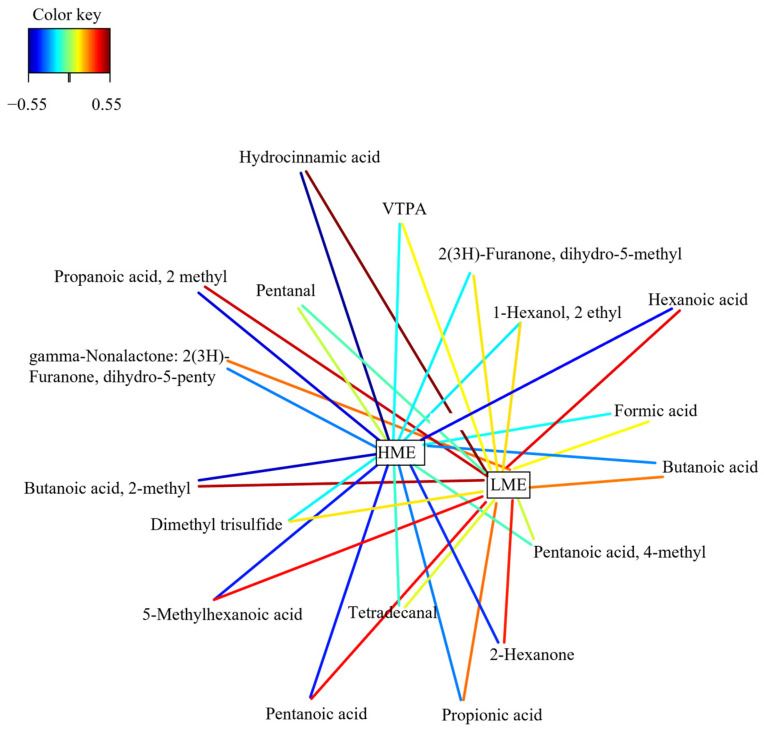
Relevance networks plot of volatile organic compounds (VOC) in the feces of low methane-emitting (LME) and high methane-emitting (HME) dairy cows. Correlation structure after Partial Least Squares Discriminant Analysis. Abbreviations: VTPA = Vitispirane A.

Association networks between the X (fecal VOC) and Y (experimental groups) datasets are shown in Figure 2. In this analysis, a positive correlation of LME with propionic acid (r = 0.43), propanoic acid, 2-methyl- (r = 0.51), butanoic acid (r = 0.43), butanoic acid, 2-methyl- (r = 0.52), hexanoic acid (r = 0.49), and hydrocinnamic acid (r = 0.55) was evident.

**Figure 3:**
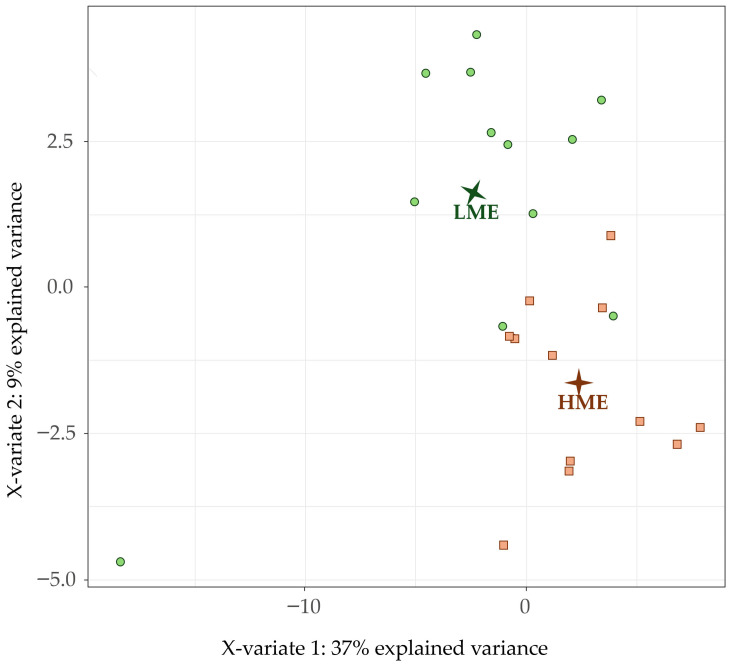
Partial Least Squares Discriminant Analysis plot of the rumen fluid volatile organic compounds of the individual dairy cows separated into the two groups: high methane-emitting (HME, brown square) and low methane-emitting (LME, green circle) dairy cows.

The PLS-DA based on all detected ruminal VOC is shown in Figure 3. The two principal components accounted for 46% of the total variance. The Y-matrix values indicate that LME and HME groups were well separated along both components and were located in opposite quadrants, suggesting opposing VOC profiles. The first component was positively associated with 3-octanol (loading: 0.14) and hexanal (loading: 0.14) and negatively associated with 2-heptanone, 5-methyl- (loading: −0.22), and sec-butyl acetate (loading: −0.20). Similarly to the fecal results, propionic acid showed a negative association on the first component (loading: −0.11), while acetic acid was weakly associated (loading: −0.08).

**Figure 4:**
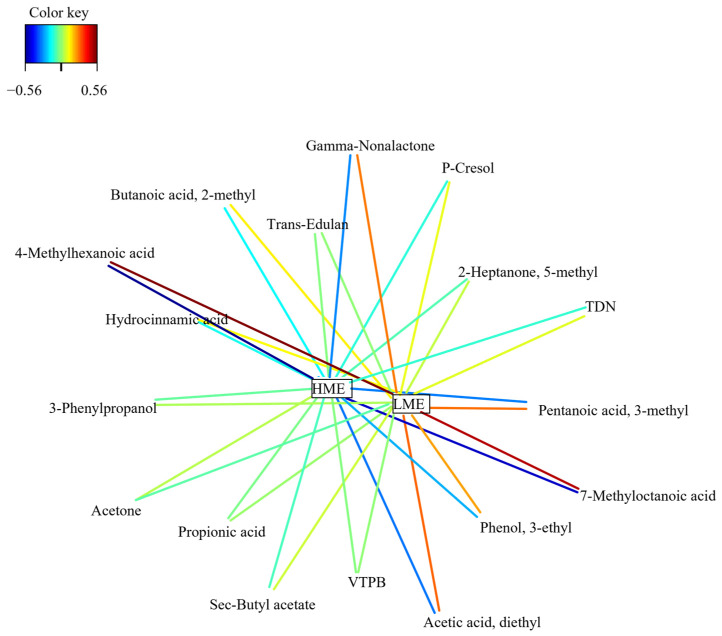
Relevance networks plot of volatile organic compounds in the rumen fluid of low me-thane-emitting (LME) and high methane-emitting (HME) dairy cows. Correlation structure after Partial Least Squares Discriminant Analysis. Abbreviations: TDN = naphthalene, 1,2-dihydro- 1,1,6-trimethyl-; VTPB = Vitispirane B.

The association networks between the X (rumen VOC) and Y (experimental groups) datasets are presented in Figure 4. This analysis shows a positive association of LME with propionic acid (r = 0.40), hydrocinnamic acid (r = 0.43), butanoic acid, 2-methyl- (r = 0.44), and pentanoic acid, 3-methyl- (r = 0.48).

### 3.3. Rumen Metagenomics

**Figure 5:**
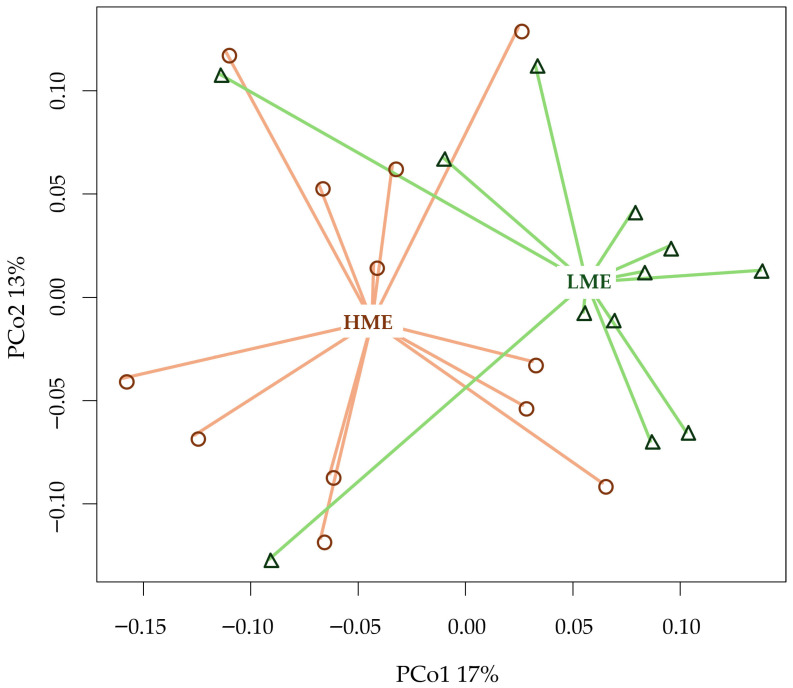
Beta diversity (Bray–Curtis distance) at order taxonomic level of high methane-emitting (HME) and low methane-emitting (LME) dairy cows using Principal Coordinates (PCo) Analysis.

The similarity among the animals in terms of the rumen microbiome composition at the order level (based on Bray–Curtis distances) is illustrated using PCoA (Figure 5), with PCo1 and PCo2 explaining 30% of the total microbial variation. The HME and LME groups were primarily separated along PCo1, which accounted for 17% of the variability. This separation was statistically significant for both β-diversity (*p* = 0.04) and permutational multivariate analysis of variance (*p* = 0.02), which considers the entire microbial composition, indicating distinct rumen microbial communities.

Table 4 presents the Shannon diversity index and microbial diversity of Archaea and key bacterial orders selected based on the highest and lowest coefficients from the permutational multivariate analysis of variance. The Shannon index revealed significantly higher microbial diversity in the LME group compared to the HME group (*p* < 0.05). Enterobacterales was higher in LME than HME animals (*p* < 0.05), while Bacteroidales and Oscillospirales were similar between the experimental groups (*p* > 0.05). Among Archaea, Methanomassiliicoccales and Methanobacteriales were detected, with only Methanobacteriales showing a significantly higher abundance in the HME group (*p* < 0.05).

**Figure 6:**
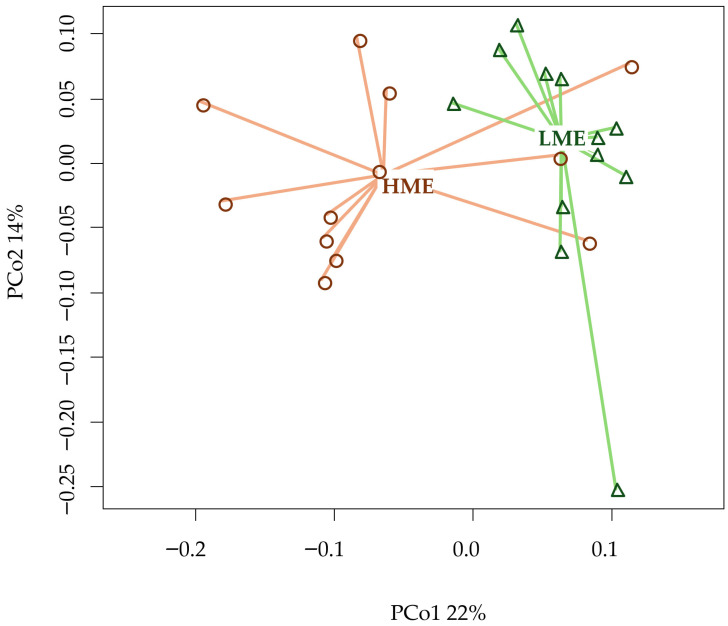
Beta diversity (Bray–Curtis distance) at the genus taxonomic level of high methane-emitting (HME) and low methane-emitting (LME) dairy cows using Principal Coordinates (PCo) Analysis.

The microbial similarity at the genus level is also shown via PCoA (Figure 6), with PCo1 and PCo2 explaining 36% of the variation in rumen microbiota. As with the order-level analysis, the groups were mostly differentiated along PCo1, which accounted for 22% of the variability. This difference was statistically significant for both β-diversity (*p* < 0.01) and permutational multivariate analysis of variance, which considers the entire microbial composition (*p* < 0.01).

Table 5 includes the Shannon index and microbial diversity of Archaea and key genera selected based on the highest and lowest coefficients from the permutational analysis. The Shannon index, as well as the relative abundance of *Prevotella*, was similar between the groups (*p* > 0.05). Conversely, *Succinivibrionaceae UCG-001* was higher (*p* > 0.05) in LME than in HME animals. Among Archaea, *Methanosphaera* (*p* < 0.05) and *Methanobrevibacter* (*p* = 0.06) were more abundant in HME animals, whereas *Candidatus Methanomethylophilus* was significantly lower in HME compared to LME animals (*p* < 0.05).

## 4. Discussion

The LME group showed significantly lower NDFd and a lower acetate/propionate ratio than the HME group. These results are in line with Stepanchenko et al. [4], who explained that reduced CH_4_ production could be linked to lower diet digestibility as a consequence of a faster rumen passage rate. Similarly, through the application of mathematical models, the authors of [34] demonstrated that a faster rumen passage rate is associated with lower CH_4_ emissions in both cows and sheep. Stepanchenko et al. [4] further hypothesized that the different passage rates were due to the smaller body size and consequently smaller rumen volume in LME cows. However, in our study, both groups belonged to the same breed and had the same BW. The lower acetate/propionate ratio observed in the LME group may be attributed to more glucogenic rumen fermentation, as supported by the higher concentrations of Succinivibrionaceae and *Selenomonas* (0.23% vs. 0.13% in the LME and HME groups, respectively; *p* < 0.01; in tables) at the genus level [35], as well as Enterobacterales at the order level. Additionally, analysis of ruminal and fecal VOC showed that the LME group was strongly associated with propionic acid and hydrocinnamic acid but less with acetic acid, further supporting this hypothesis.

The production of propionic acid consumes two moles of H_2_ per mole of glucose, thus reducing the availability of H_2_ for methanogenesis [9]. Therefore, a lower acetate/propionate ratio, favoring propionate, serves as an indicator of the rumen microbiota’s ability to support energy-efficient fermentation, with reduced CH_4_ emissions [36]. Guo et al. [37] explained that hydrocinnamic acid originates from ruminal cellulose metabolism and is downregulated when fiber digestibility decreases. Auffret et al. [38] discussed how populations of Proteobacteria, of which Enterobacteriaceae are key members, tend to increase with propionate synthesis in the rumen, thereby reducing the availability of H_2_ for methanogenesis. According to Granja-Salcedo et al. [39], supplementation with encapsulated nitrate increased the abundance of Proteobacteria, including Enterobacteriaceae. These bacteria use fumarate as an electron acceptor and compete with methanogens for H_2_, potentially reducing CH_4_ production. This may have led to more energy being available to rumen microbes in the LME group [40], thus potentially increasing the protein and nitrogen utilization at the rumen level. However, this additional energy was likely only sufficient to numerically, but not significantly, increase milk production in LME compared to HME.

The rumen and feces VOC analysis revealed that LME was positively associated with butanoic acid, 2-methyl-, a branched-chain VFA that, as reported by Zhang et al. [41], derives from amino acid degradation and serves as an important cofactor for the growth of microorganisms such as *Ruminococcus* (5.4% vs. 3.6%, *p* = 0.02) and *Fibrobacter* (4.0% vs. 2.4%, *p* = 0.04), both of which were more abundant in LME rumen fluid than in HME. However, since both *Fibrobacter* and *Ruminococcus* are fibrolytic bacteria [9], their higher abundance in LME, where the NDFd was lower, could be explained by a faster rumen passage rate. The mechanism of fiber digestion in the rumen constitutes a dynamic balance between the fiber’s rate of digestion and the passage rate of the digesta through the rumen [42]. In a study on the feeding behavior of Italian Simmental and Holstein Friesian cows, Florit et al. [43] associated shorter ET with higher NDFd. However, in our study, we speculate that the potential difference in the passage rate was not due to the animal size or feeding behavior, as the ET and RT were similar between groups, but may be due to neuroendocrine differences affecting rumen motility [44]. Furthermore, this possible variation could influence enteric CH_4_ production. Indeed, a previous study [45] established a strong inverse relationship between the amount of CH_4_ yield and the passage rate of digesta.

Overall, the two experimental groups differed clearly in terms of the VOC and the microbiota of both feces and, more prominently, the rumen. The results of β-diversity analysis indicated that the overall taxonomic composition at the genus and order levels differed between groups. Furthermore, the Shannon diversity index (α-diversity) values revealed that animals with higher CH_4_ production had lower microbial diversity at the order level. This agrees with previous findings showing that higher microbial diversity in the rumen is associated with lower CH_4_ production. In particular, Conteville et al. [46] observed that cows with lower CH_4_ emissions had higher microbial diversity in both the feces and rumen. As previously explained, microbial communities associated with low-emission animals showed enhanced carbohydrate metabolic activity, suggesting a link between microbial diversity, digestive efficiency, and CH_4_ reduction. Furthermore, increased microbial diversity may correspond to increased competition with methanogenic archaea for hydrogen utilization [47]. Tapio et al. [48] reported that animals with lower CH_4_ emissions had more diverse microbial communities, characterized by a reduced abundance of methanogenic archaea and H_2_-producing microbes.

In our study, HME animals had a higher abundance of Methanobacteriales at the order level, the most significant methanogenic group in the rumen, including *Methanobrevibacter* and *Methanosphaera* at the genus level [9], both of which were significantly or numerically higher in HME than in LME. These results align with Wallace et al. [7], who found that *Methanobrevibacter* and *Methanosphaera* were 2.5× and 2.4× more abundant, respectively, in animals emitting high levels of CH_4_. In contrast, no significant differences in Methanomassiliicoccales at the order level were observed in our study. This differs from Danielsson et al. [49], who found higher levels of Methanomassiliicoccales in low-emitting cows. However, the genus *Candidatus Methanomethylophilus* was found in much higher concentrations in LME cows, in agreement with the findings of Auffret et al. [38]. In general, it should be considered that CH_4_ formation in the rumen depends not only on the abundance of methanogens but also on their diversity. In addition to dietary and environmental factors, host genetics can also determine this diversity by influencing hydrogen availability in the rumen and the interactions between H_2_-producing microbes [50].

However, although extreme CH_4_-emitter dairy cows were considered to ensure adequate power for the statistical test, the HME group showed higher variability in methane emissions than the LME group. Consequently, caution should be used when extrapolating these findings to the general population.

## 5. Conclusions

This trial underlined that there was a clear difference between the high- and low-emitting cows. The higher CH_4_ production of HME cows appears to stem from variations in the rumen microbiota composition, with a predominance of methanogenic bacteria and a lower presence of H_2_-competing bacteria, thereby reducing methanogenesis. The difference in microbial populations was associated with a shift in the acetate/propionate ratio and NDFd, which in this case appear to be independent of animal size or feeding behavior. The metabolic profiles of the two groups suggest that CH_4_ mitigation in dairy cows could benefit from interventions targeting the rumen microbiota, aiming to make it more diverse, resilient, and dominated by bacterial communities that compete for H_2_. These findings underscore the potential of strategies focused on the ruminal microbiome to reduce methane emissions, pointing toward a practical approach based on the use of microbiome data in animal selection. Prioritizing individuals that naturally host microbial communities less oriented toward methanogenesis and more competitive for hydrogen and incorporating this criterion into herd-level decision making may contribute to lowering emissions while maintaining energy-efficient rumen fermentation.

## Figures and Tables

**Table 1:** Feedstuff and nutrients composition of the total mixed ratio based on a dry matter basis.

Items	Values
Feedstuff, % DM	
Italian ryegrass silage	26.4
Alfalfa silage	20.6
Corn meal	16.6
Whole-ear corn silage	15.9
Soybean cake	15.8
Mineral-vitamin premix	2.04
Beet pulp	1.92
Sodium bicarbonate	0.60
DM, % of fresh matter	52.1
Nutrients, % DM	
CP	14.9
EE	3.10
NDF	35.8
ADF	18.9
Ash	8.10
Energy content, UFL	0.90

DM = dry matter; CP = crude protein; EE = ether extracts; NDF = neutral detergent fiber; ADF = acid detergent fiber; UFL = net energy for lactation calculated according to INRA standard [16].

**Table 2:** Performance, feeding behavior, diet components digestibility and methane emissions of high methane-emitting (HME) and low methane-emitting (LME) dairy cows. Data are expressed as the median (interquartile range).

	Group		
Item	HME	LME	*p*-Value
Performance			
FCM (kg/day)	29.0 (6.7)	34.3 (5.9)	0.07
Milk fat (%)	3.78 (0.48)	3.51 (0.35)	0.05
Milk protein (%)	3.41 (0.23)	3.26 (0.17)	0.17
DMI (kg/day)	22.1 (2.1)	23.2 (3.0)	0.22
Behavior			
ET (min/day)	162.2 (77.6)	174.2 (76.7)	0.49
RT (min/day)	524.0 (50.9)	514.5 (96.3)	0.71
Digestibility (%)			
DM	71.6 (4.1)	70.8 (2.8)	0.32
CP	70.0 (3.4)	60.5 (5.3)	0.63
NDF	51.4 (4.6)	47.9 (6.6)	0.04
ADF	45.9 (6.1)	46.3 (6.1)	0.24
Methane production (g/kg)			
CH_4_/DMI	22.5 (11.6)	13.2 (3.2)	<0.01
CH_4_/FCM	16.9 (10.5)	8.4 (2.6)	<0.01

FCM = fat corrected milk; DMI = dry matter intake; ET = eating time; RT: rumination time; DM = dry matter; CP = crude protein; NDF = neutral detergent fiber; ADF = acid detergent fiber; CH_4_ = methane.

**Table 3:** Feces and rumen fermentation variables of high methane-emitting (HME) and low methane-emitting (LME) dairy cows. Data are expressed as the median (interquartile range).

	Group		
Item	HME	LME	*p*-Value
Faeces			
pH	6.96 (0.35)	6.80 (0.35)	0.22
NH_3_ (mg/L)	20.0 (15.0)	22.6 (11.3)	0.14
Lactic acid (mmol/L)	0.358 (0.490)	0.068 (0.329)	0.33
Acetic acid (mmol/L)	2.75 (0.68)	2.69 (0.41)	0.82
Propionic acid (mmol/L)	0.431 (0.131)	0.455 (0.055)	0.23
Isobutyric acid (mmol/L)	2.88 (2.01)	1.24 (2.40)	0.31
Butyric acid (mmol/L)	0.204 (0.099)	0.218 (0.050)	0.97
Valeric acid (mmol/L)	0.165 (0.203)	0.113 (0.104)	0.30
Acetic/Propionic acid	6.45 (2.13)	5.60 (1.17)	0.16
Rumen fluid			
pH	7.11 (0.68)	6.70 (0.61)	0.21
NH_3_ (mg/L)	7.00 (4.04)	8.03 (4.73)	0.97
Lactic acid (mmol/L)	0.000 (0.007)	0.007 (0.038)	0.18
Acetic acid (mmol/L)	2.76 (0.73)	2.85 (0.93)	0.91
Propionic acid (mmol/L)	0.761 (0.204)	0.835 (0.281)	0.41
Isobutyric acid (mmol/L)	0.038 (0.015)	0.034 (0.013)	0.37
Butyric acid (mmol/L)	0.433 (0.127)	0.596 (0.315)	0.16
Isovaleric acid (mmol/L)	0.067 (0.033)	0.071 (0.040)	0.28
Valeric acid (mmol/L)	0.089 (0.095)	0.060 (0.024)	0.04
Acetic/Propionic acid	3.53 (0.226)	3.31 (0.188)	<0.01

NH_3_: Ammonia.

**Table 4:** Rumen microbial diversity and microbial composition in high methane-emitting (HME) and low methane-emitting (LME) dairy cows at the order taxonomic level. Data are expressed as the median (interquartile range).

	Group		
Item	HME	LME	*p*-Value
Shannon Index	1.95 (0.14)	2.08 (0.11)	0.02
Order taxa (%) ^1^			
Enterobacterales	1.30 (2.52)	3.77 (4.95)	0.03
Bacteroidales	41.91 (8.88)	38.47 (5.66)	0.11
Oscillospirales	16.74 (4.02)	15.74 (4.91)	0.16
Archea order taxa (%)			
Methanobacteriales	0.10 (0.10)	0.03 (0.04)	0.03
Methanomassiliicoccales	0.01 (0.04)	0.03 (0.06)	0.61

^1^ Microbial order taxa with the highest and lowest coefficients (>|1|) after permutational multivariate analysis of variance.

**Table 5:** Rumen microbial diversity and microbial composition in high methane-emitting (HME) and low methane-emitting (LME) dairy cows at the genus taxonomic level. Data are expressed as the median (interquartile range).

	Group		
Item	HME	LME	*p*-Value
Shannon Index	3.43 (0.20)	3.41 (0.19)	0.70
Genus taxa (%) ^1^			
*Succinivibrionaceae UCG-001*	0.13 (0.33)	1.28 (2.36)	0.04
*Prevotella*	23.11 (8.09)	22.56 (4.34)	0.25
Archea genus taxa (%)			
*Methanosphaera*	0.02 (0.03)	0.00 (0.00)	0.04
*Candidatus Methanomethylophilus*	0.00 (0.00)	0.01 (0.04)	0.04
*Methanobrevibacter*	0.07 (0.10)	0.02 (0.02)	0.06

^1^ Microbial genus taxa with the highest and lowest coefficients (>|1|) after permutational multivariate analysis of variance.

## Data Availability

All data are available from the corresponding author on reasonable request.
